# Crossmodal Audiovisual Emotional Integration in Depression: An Event-Related Potential Study

**DOI:** 10.3389/fpsyt.2021.694665

**Published:** 2021-07-20

**Authors:** Ting Lu, Jingjing Yang, Xinyu Zhang, Zihan Guo, Shengnan Li, Weiping Yang, Ying Chen, Nannan Wu

**Affiliations:** ^1^Department of Psychology, Faculty of Education, Hubei University, Wuhan, China; ^2^School of Artificial Intelligence, Changchun University of Science and Technology, Changchun, China

**Keywords:** ERPs, audiovisual integration, multisensory processing, depression, emotion

## Abstract

Depression is related to the defect of emotion processing, and people's emotional processing is crossmodal. This article aims to investigate whether there is a difference in audiovisual emotional integration between the depression group and the normal group using a high-resolution event-related potential (ERP) technique. We designed a visual and/or auditory detection task. The behavioral results showed that the responses to bimodal audiovisual stimuli were faster than those to unimodal auditory or visual stimuli, indicating that crossmodal integration of emotional information occurred in both the depression and normal groups. The ERP results showed that the N2 amplitude induced by sadness was significantly higher than that induced by happiness. The participants in the depression group showed larger amplitudes of N1 and P2, and the average amplitude of LPP evoked in the frontocentral lobe in the depression group was significantly lower than that in the normal group. The results indicated that there are different audiovisual emotional processing mechanisms between depressed and non-depressed college students.

## Introduction

Depression is a common mood disorder characterized by depressive mood, anhedonia and cognitive deficits (1). Approximately 8% of men and 15% of women are likely to suffer from depression in their lifetime (2). As an affective disorder with high morbidity, recurrence and disability rates, depression poses a serious threat to human physical and mental health, so it has always been an important subject of human research (3). Depression is related to defects in emotion processing, which manifest as deviations in emotion perception and dysfunction in psychological mechanisms related to emotion regulation (4, 5). Specifically, compared with healthy individuals, depressed individuals are more sensitive to negative emotional stimuli but less sensitive to positive emotional stimuli, which are reflected in perception, attention and memory (6). In addition, depressed individuals' processing of neutral stimuli is different from that of healthy individuals. According to the emotion context insensitivity model, depressed individuals are more sensitive to neutral emotional stimuli than normal individuals (7). Other studies have shown that those with depression are more likely to perceive neutral faces as sad faces because depression affects the perception of negative stimuli (8, 9).

At the neurobiological level, there are also a large number of studies exploring the relationship between depression and emotional processing defects. For example, a magnetic resonance imaging (MRI) study showed that participants with a history of depression had a significant attentional bias to negative emotional stimuli (10), and negative attentional bias may be one of the key factors of depression recurrence (11). A study using event-related potential (ERP) technology examined the late positive potential (LPP) evoked in depressed participants when they processed sad pictures and happy pictures, and there was no significant difference in the amplitude of LPP (12). However, in the study of healthy subjects, it was found that the amplitude of LPP induced by sad pictures was significantly larger than that induced by happy pictures (13), and negative emotional stimulation could induce stronger electrophysiological responses (14–16), so subjects may devote more attention to negative emotional stimuli, thus realizing the fine coding of emotional stimuli. The difference of LPP amplitude of positive and negative emotional stimuli between depressed patients and healthy subjects indicates that the fine processing ability of depressed participants to negative emotional stimuli is weakened, or negative information may not be able to induce strong emotional and motivational responses in depressed individuals (12). At the same time, the research on the emotional sound of depression subjects also shows that the depression group has a very low degree of arousal to negative emotional sound stimulation, but the physiological response is very strong, which makes the autonomic nervous system in an abnormal state for a long time, which can easily lead to physical discomfort (12). In addition, compared with controls, depressed patients showed relatively high N2 and reduced P3 amplitudes to negative compared with positive target stimuli, as well as marginally reduced N2 amplitude to positive target stimuli (17).

However, most of the above was the result of an individual's single-channel emotional stimuli processing, that is, judging others' emotions through visual (such as observing facial expression) or auditory (such as analyzing tone) stimuli, but in daily life, people engage in crossmodal emotional processing. A large number of studies have shown that an individual's single-channel processing results may be affected by other channels (18–22); therefore, it is inappropriate to generalize the results of research on a single channel directly to the understanding of people's daily lives. The underlying neurocognitive processes that integrate separate streams of information from different sensory channels into the overall experience are often referred to as multisensory integration (MSI) (23). Researchers focused on healthy adults (18), patients with bipolar disorder (24), and patients with schizophrenia (25, 26) showed that multisensory integration of emotional cues is beneficial because the integration of information from multiple channels will increase the recognition speed and hit rate of emotional information.

For people with depression, although researchers agree that there may be problems in the process of emotional information recognition, there is a lack of a more ecological exploration of crossmodal emotional integration processing ability. Therefore, this study will adopt the perceptual crossmodal integration of auditory and visual stimuli and use ERP technology to study the effects of cross-channel integration of emotional information in depressed people to reveal the characteristics of crossmodal audiovisual emotional integration in depressed individuals and provide an experimental basis for the pathogenesis of depression. In previous studies on crossmodal integration of emotion, N1 and P2 were the two main components affected by the integration of visual and vocal emotional information (27, 28). N1 is associated with stimulus detection (29). It has been found that the amplitude of N1 induced by cross-channel emotional stimulation with consistent audiovisual emotional valance is lower than that of a single audio channel. N1 inhibition is considered a specific indicator of the integration of two-channel audiovisual emotional information (30, 31). However, other studies have found that consistent dual-channel audiovisual emotional information induces stronger N1 amplitudes than single-channel emotional information (32). P2 is an important indicator for the rapid detection of emotional stimuli (31, 33–35). Researchers believe that N1 and P2 are a reflection of visual and audio emotional information being automatically integrated within 200 ms, which represents the processing of low-level visual features of emotional stimuli (36). Therefore, previous studies used N1 and P2 as important indicators of crossmodal emotional integration. In addition, we also analyzed N2 and LPP components to test whether depression affects the processing of emotional information integration. The N2 component reflects the active inhibition of negative stimuli (37), and LPP components reflect the high-level cognitive processing of emotion (38). Therefore, we hypothesized that the amplitudes of the N1, P2, N2, and LPP components would be relatively larger in the depression group than in the normal group.

## Methods And Materials

### Participants

Forty-six college students from a university in Hubei participated in the experiment, including 23 in the healthy normal group (10 males and 13 females; age: 19.83 ± 1.30 years; depression score: 6.74 ± 2.83) and 23 in the depression group (10 males and 13 females; age: 20.14 ± 1.17 years; depression score: 24.78 ± 5.26). Both groups had normal hearing and visual or corrected visual acuity and were right-handed. Each subject signed informed consent before entering the study. The screening methods were as follows: First, the Beck Depression Inventory-II (BDI-II) questionnaire was distributed online (https://www.wjx.cn/) to undergraduates in a university in Hubei Province, China. A total of 352 people submitted the questionnaire; after excluding those with incomplete information and scale items, 337 questionnaires remained. College students with BDI-II scores ≥19 were selected as the depression group, and college students with BDI-II scores ≤ 13 were selected as the healthy control screening group (12). Then, within 1–2 weeks, 47 college students (24 in the depression group and 23 in the healthy normal group) were invited to participate in the structured interview by telephone. Based on the Structured Clinical Interview for DSM (SCID), one depressed patient who was taking medication was excluded after the interview. Finally, 23 people were enrolled in the depression group and 23 people in the healthy normal group. The participants provided written informed consent to participate in this study, which was previously approved by the Ethics Committee of Hubei University. All participants received payment for their time.

### Stimuli

The visual materials for this experiment were selected from the Chinese Affective Face Picture System (CAFPS), and we selected 10 happy, 10 neutral and 10 sad pictures of emotional faces (half male and half female), a total of 30. Audio materials were selected from The Montreal Affective Voice (MAV), and we selected 10 happy, 10 neutral and 10 sad emotional voices, a total of 30. Fifty college students were invited in advance to evaluate the valence and arousal of the visual and audio materials on a 7-point rating scale (1 means very sad; 4 means neutral; 7 means very happy). The evaluation results were compared with a score of 4 by single sample *t*-tests. Three happy pictures and two sad pictures showing no significant differences were excluded.

Considering that the subjects of this experiment include special people, people with depression are less sensitive to emotion, in order to make people with depression have a higher level of emotional arousal and avoid the influence of gender factors on this experiment, finally, one picture with the highest average score and one voice with the highest average score were selected from the happy emotion pictures and voices that met the requirements (female face and voice). Select the one with the lowest average score from the required sad pictures and sounds and the experimental material with a sound of sadness (female faces and voices). The final evaluation results of the materials are shown in Table 1.

**Table 1:** Mean evaluation scores for the selected experimental materials.

**Materials**	**Emotion**	***M***	***SD***
Face	Sad	2.24	1.28
	Neutral	3.90	0.37
	Happy	6.93	0.73
Voice	Sad	1.80	0.39
	Neutral	3.91	0.51
	Happy	6.82	0.67

### Procedure

**Figure 1:**
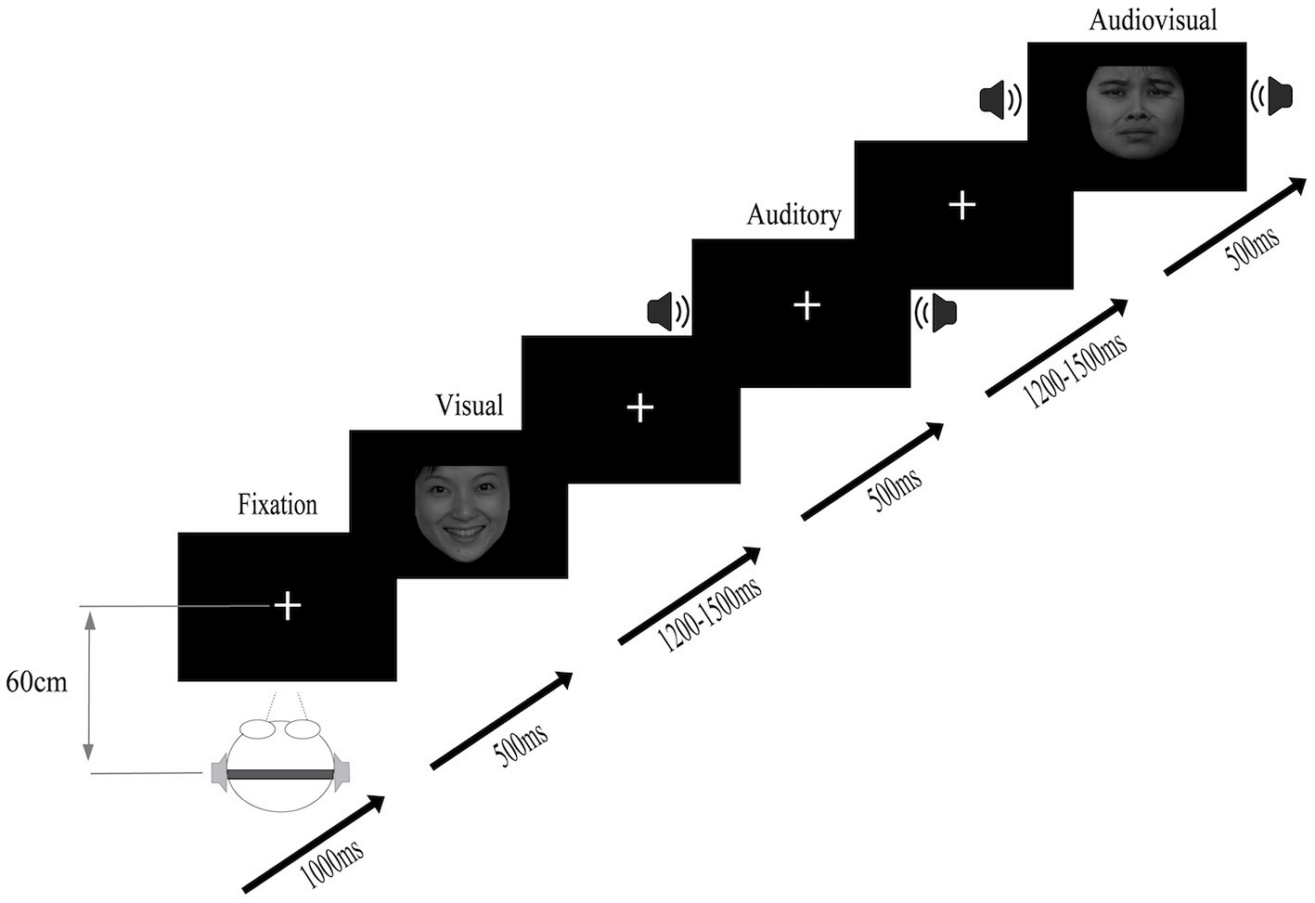
Stimuli were randomly presented in audio, visual, and audiovisual conditions, and participants were asked to respond to target stimuli (auditory target, visual target, and audiovisual target).

The experiment was carried out in a quiet, soundproof laboratory at an appropriate temperature. The participants sat in front of a 15.6-inch color LCD (screen resolution was 1,024 × 768 pixels, refresh rate was 100 Hz), and the distance between the participants and the screen was ~60 cm. The experimental program was developed by E-Prime 2.0 (Psychology Software Tools, Inc., Pittsburgh, PA, USA). During the experiment, as shown in Figure 1, the participants fixated on a cross on the screen. The participants' task was to respond to the target stimuli as quickly and accurately as possible using their two hands on the keyboard (press “S” for sad, “G” for neutral, and “K” for happy; the keys setting makes the balance between the subjects), regardless of whether an auditory/visual/audiovisual stimulus was presented. The stimulus stream consisted of a visual stimulus, an auditory stimulus and an audiovisual stimulus, all presented using Presentation software (Neurobehavioral Systems Inc., Albany, California, USA). The sequence of the stimuli was randomized across different streams. Each participant needed to finish 5 blocks, and each block contained 720 trials. After finishing each block, they were allowed to rest.

### Apparatus

The behavioral and electroencephalographic data were recorded simultaneously. Stimulus presentation was controlled using E-prime 2.0, and participants were asked to respond via the keyboard. The auditory stimuli were presented through an earphone. Electroencephalography (EEG) signals were recorded by an EEG system (BrainAmp plus, Gilching, Germany) through 32 electrodes (Easy-cap, Herrsching–Breitbrunn, Germany). Horizontal eye movements were detected using an electro-oculogram (EOG) electrode placed at the outer canthi of the left eye, and vertical eye movements and blinks were measured by the EOG from one electrode positioned ~1 cm below the participant's right eye. Impedance was maintained below 5 kΩ from all electrodes. All electrodes were referenced to the FCz electrode and were rereferenced offline to the average of both mastoids. The EEG was digitized at a sampling rate of 500 Hz.

### Data Analysis

#### Behavioral Data Analysis

The collected experimental data were preprocessed and eliminated based on the following criteria: (1) participants whose average hit rate was lower than 80% and (2) participants who made error responses. Data from 20 participants in the depression group and 22 participants in the normal group were eventually included in the analysis. The average response time of each participant under each treatment condition was calculated, and the data exceeding plus or minus three standard deviations from the mean were deleted to obtain the average response time. To analyze the hit rate and response time (RT) of the two groups, we performed 2 (group: depression, control) × 3 (modality: visual, audio, audiovisual) × 3 (emotion: happy, neutral, sad) mixed-factors ANOVA with group as a between-subject factor and the other two variables as within-subject factors. The Greenhouse-Geisser method was used if necessary to correct for non-sphericity. The statistical significance level was set at *p* < 0.05 (Mauchly's sphericity test). The effect size estimates $\eta_{p}^{2}$ are reported.

#### ERP Data Analysis

The EEG data were analyzed by using Brain Vision Analyzer software (Version 2.0, Brain Products GmbH, Munich, Bavaria, Germany). Only the trials in which performance was correct were used for further analyses. All signals were referenced to both mastoid processes (TP9/TP10). EEG signals were bandpass filtered with a range of 0.01–60 Hz, divided into epochs (80 audiovisual epochs, 80 visual epochs, 80 auditory epochs for each emotion, 720 epochs in total) from 100 ms before stimulus onset to 800 ms after stimulus onset. Baseline correction was then performed with the signal measured from −100 ms to 0 ms relative to stimulus onset. The artifact correction was performed by rejecting signals that exceeded ± 100 μV. An overall average was obtained from the data for each stimulus type. In addition, bandpass filtering was carried out again at the standard of 0.3–30 Hz, and baseline correction was performed with the signals from −100 to 0 ms. The grand-averaged data were obtained for each stimulus type at each electrode in each group with each emotions. Participants who lost more than 70% of the epochs of one type of stimulus were excluded.

The difference wave [AV-(A+V)] was calculated as the effect of audiovisual integration (39, 40). In other words, audiovisual integration was the difference between the ERPs to bimodal (AV) stimuli and the ERPs to the sum of the unimodal stimuli (A+V) (41–45). The ERP components analyzed in the present study included N1, P2, N2, and LPP. Component amplitudes and latencies were recorded at the component peaks. N1 was scored as the maximum negative amplitude in the time window from 120 to 140 ms after stimulus onset; P2 was scored as the most positive amplitude in the time window from 160 to 200 ms; N2 was scored as the most negative peak in the time window from 300 to 340 ms; and LPP was scored as the most positive peak in the time window from 380 to 420 ms. Three regions of interest (frontocentral region: FC1, FC2, Fz; parietal region: P3, P4, Pz; central region: C3, C4, Cz) were selected for further analysis. Mixed-measures analysis of variance (ANOVA) was conducted on each ERP component with group as a between-subject factor and emotion and regions of interest (ROIs) as within-subject factors. The Greenhouse-Geisser adjustment was applied to the degrees of freedom of the F ratios as necessary. All statistical analyses were carried out using SPSS 21.0.

## Results

### Behavioral Results

The response time and hit rate of the participants under various conditions are shown in Table 2.

**Table 2:** Mean response times (ms) and hit rates (%) in both the depression and normal groups in response to different emotions.

	**Visual**			**Auditory**			**Audiovisual**		
	**Sad**	**Neutral**	**Happy**	**Sad**	**Neutral**	**Happy**	**Sad**	**Neutral**	**Happy**
**Depression group (*****N*** **=** **20)**									
RT	593.40 (68.19)	559.22 (60.53)	607.76 (74.26)	706.77 (104.95)	640.32 (94.17)	700.65 (124.75)	574.03 (68.78)	537.31 (58.52)	605.26 (89.43)
Hit rate	94.75 (4.01)	96.44 (2.54)	95.38 (4.06)	91.94 (7.95)	96.63 (2.96)	93.25 (5.48)	97.69 (2.82)	98.19 (1.70)	97.56 (2.55)
**Normal group (*****N*** **=** **22)**									
RT	682.88 (106.85)	623.95 (80.08)	660.32 (85.54)	793.25 (131.31)	696.23 (115.98)	783.27 (147.34)	673.64 (112.86)	598.20 (88.76)	656.95 (104.55)
Hit rate	93.13 (5.96)	96.31 (2.79)	94.20 (4.62)	90.97 (8.42)	93.98 (4.53)	88.98 (8.07)	96.25 (2.75)	97.61 (3.53)	94.55 (4.83)

*Standard deviations are given in parentheses*.

#### Hit Rate

The hit rate was analyzed by three-factor mixed ANOVA with 2 (group: depression group, normal group) ^*^ 3 (emotion: sad, neutral, happy) ^*^ 3 (modality: visual, auditory, audiovisual) factors. Among them, group is a between-subject factor, and emotion and modality are within-subject factors. The results showed that the main effect of modality was significant, *F*_(2, 80)_ = 29.01, *p* < 0.001, $\eta_{p}^{2}$ = 0.42. The hit rate with the audiovisual modality (97%) was significantly higher than that with the visual modality (95%) and auditory modality (93%), which shows the advantage of crossmodal processing. The main effect of emotion was significant, *F*_(2, 80)_ = 14.01, *p* < 0.001, $\eta_{p}^{2}$ = 0.259. Further analysis showed that the hit rate with happy emotional stimuli was significantly higher than that with sad and neutral emotional stimuli (all *p* < 0.001). There was no significant difference between the hit rate with sad and neutral emotional stimuli. The main effect of group was not significant, *F*_(1, 40)_ = 3.03, *p* = 0.089, $\eta_{p}^{2}$ = 0.07. The hit rate in the depression group (96%) was not significantly different from that in the normal group (94%). The three-way interaction was not significant, *F*_(4, 160)_ = 1.16, *p* = 0329, $\eta_{p}^{2}$ = 0.028. The interaction between modality and group was not significant, *F*_(2, 80)_ = 0.88, *p* = 0.390, $\eta_{p}^{2}$ = 0.022. The interaction between emotion and group was not significant, *F*_(2, 80)_ = 1.56, *p* = 0.217, $\eta_{p}^{2}$ = 0.038. The interaction between modality and emotion was significant, *F*_(4, 160)_ = 3.04, *p* = 0.035, $\eta_{p}^{2}$ = 0.071. Further simple effect analysis showed that the hit rate with happy emotional stimuli was significantly higher than that with neutral and sad emotional stimuli under visual, auditory and audiovisual conditions (*p* < 0.001). There were no differences between neutral emotional stimuli and sad emotional stimuli (*p* > 0.05).

#### Response Time

The mixed ANOVA for response time with 2 (group: depression group, normal group) ^*^ 3 (emotion: sad, neutral, happy) ^*^ 3 (modality: visual, auditory, audiovisual) factors was carried out. The results showed that the main effect of modality was significant, *F*_(2, 80)_ = 165.85, *p* < 0.001, $\eta_{p}^{2}$ = 0.806. Further analysis showed that the response time for participants to audiovisual stimuli (*M* = 608 ms, *SD* = 13 ms) was significantly faster than that to single-channel visual stimuli (*M* = 621 ms, *SD* = 12 ms, *p* = 0.001) and auditory stimuli (*M* = 720 ms, *SD* = 18 ms, *p* < 0.001), indicating that there was a certain dominant effect in crossmodal processing. It took the longest time to respond to an emotion in the auditory channel (*p* < 0.001). The main effect of emotion was significant, *F*_(2, 80)_ = 51.49, *p* < 0.001, $\eta_{p}^{2}$ = 0.563. The participants' response to happiness (*M* = 609 ms, *SD* = 13 ms) was significantly faster than that to sadness (*M* = 671 ms, *SD* = 15 ms) and neutral (*M* = 669 ms, *SD* = 16 ms) emotional stimuli (all *p* < 0.001). There was no significant difference in response times between sad and neutral emotional stimuli (*p* = 0.744). The main effect of group was significant, *F*_(1, 40)_ = 6.55, *p* = 0.014, $\eta_{p}^{2}$ = 0.141. The response time in the depression group (*M* = 614 ms) was significantly lower than that in the normal group (*M* = 685 ms). The three-way interaction was not significant, *F*_(4, 160)_ = 2.51, p = 0.075, $\eta_{p}^{2}$ = 0.002. The interaction between modality and group was not significant, *F*_(2, 80)_ = 0.08, *p* = 0.82, $\eta_{p}^{2}$ = 0.141. There was a marginally significant interaction between emotion and group, *F*_(2, 80)_ = 3.09, *p* = 0.055, $\eta_{p}^{2}$ = 0.072. The interaction between modality and emotion was significant, *F*_(4, 160)_ = 6.17, *p* = 0.002, $\eta_{p}^{2}$ = 0.134. A simple effect analysis showed that under visual, auditory, and audiovisual conditions, the responses of participants to happy emotional stimuli were significantly faster than those to neutral and sad emotional stimuli (*p* < 0.001). There were no differences between neutral emotional stimuli and sad emotional stimuli (*p* > 0.05).

### ERP Results

#### N1 Component

**Figure 2:**
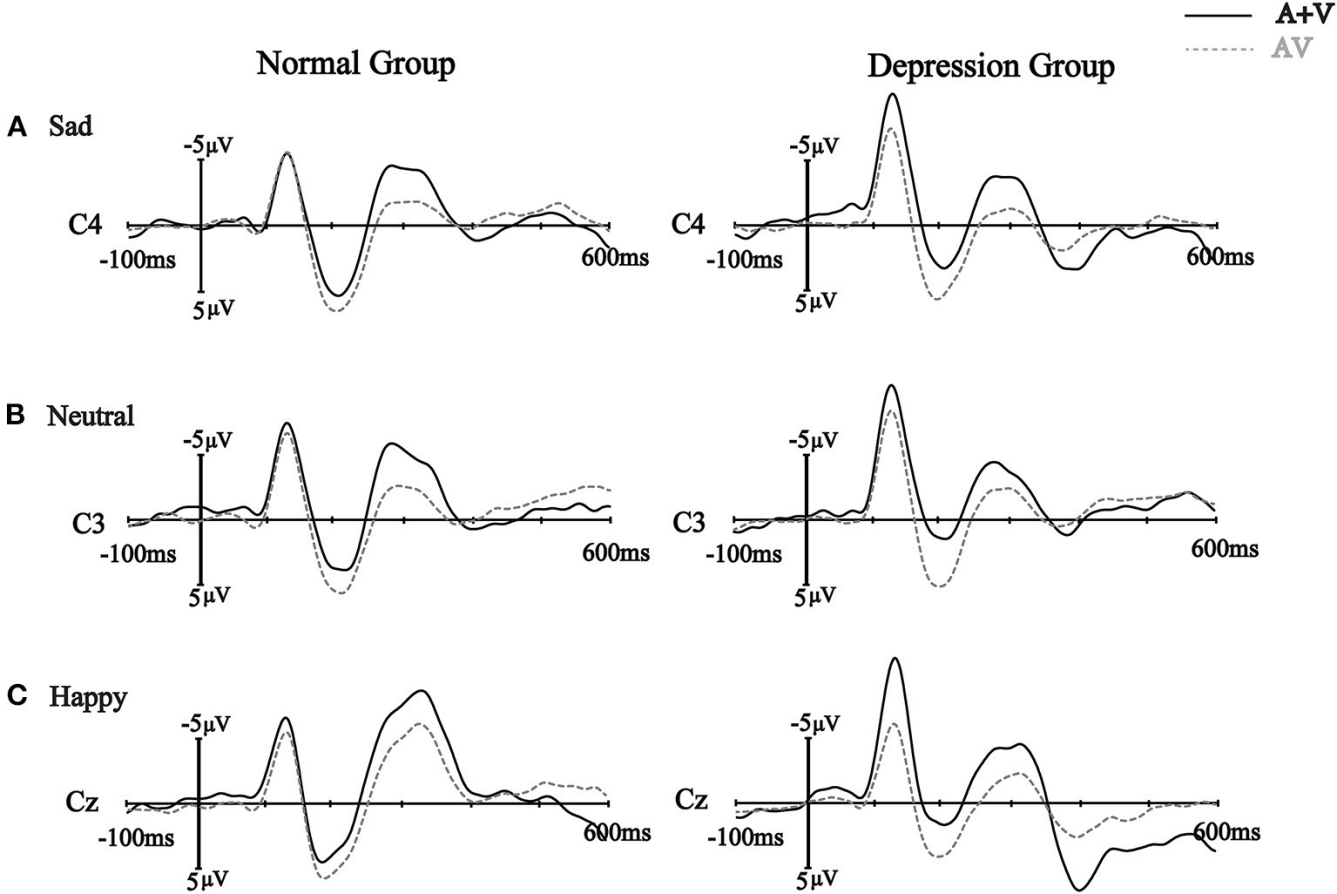
The sum of the event-related potentials of the unimodal stimuli (A + V) and the event-related potentials of the bimodal stimuli (AV) from a subset of electrodes are shown from 100ms before to 600 ms after stimulus onset in the different groups with the different emotional stimuli **(A)** sad emotional stimuli, **(B)** neutral emotional stimuli and **(C)** happy emotional stimuli.

In the 120–140 ms interval, the amplitude of ERP(AV) - [ERP(A) +ERP(V)] was analyzed using a 2 (group: depression group, normal group) ^*^ 3 (emotion: sad, neutral, happy) ^*^ 3 (ROI: parietal lobe, central lobe, frontocentral lobe) mixed-factors ANOVA with group as a between-subject factor and the other two variables as within-subject factors. The results showed that the main effect of emotion was not significant, *F*_(2, 80)_ = 0.27, *p* = 0.74, $\eta_{p}^{2}$ = 0.007. The main effect of group was significant, *F*_(1, 40)_ = 5.52, *p* = 0.02, $\eta_{p}^{2}$ = 0.121. Further analysis showed that N1 amplitude in the depression group (*M* = 1.65 μV) was higher than that in the normal group (*M* = 0.51 μV; *p* = 0.02). The main effect of ROI was significant, *F*_(2, 80)_ = 26.22, *p* < 0.001, $\eta_{p}^{2}$ = 0.396. The average N1 amplitude in the frontocentral lobe (*M* = 1.81 μV) was significantly higher than that in the central lobe (*M* = 1.20 μV; *p* < 0.001) and the parietal lobe (*M* = 0.23 μV; *p* < 0.001). The three-way interaction was significant, *F*_(4, 160)_ = 3.70, *p* = 0.02, $\eta_{p}^{2}$ = 0.085. The interaction between emotion and group was not significant, *F*_(2, 80)_ = 0.68, *p* = 0.50, $\eta_{p}^{2}$ = 0.017, and the interaction between group and ROI was not significant, *F*_(2, 80)_ = 2.20, *p* = 0.14, $\eta_{p}^{2}$ = 0.052. However, the interaction between emotion and ROI was significant, *F*_(4, 160)_ = 8.05, *p* < 0.001, $\eta_{p}^{2}$ = 0.168. Simple effect analysis showed that N1 amplitudes in the frontocentral lobe induced by the three kinds of emotional stimuli (neutral: *M* = 2.02 μV, happy: *M* = 2.07 μV, sad: *M* = 1.28 μV) were significantly larger than those in the central lobe (happy: *M* = 1.33 μV, neutral: *M* = 1.41 μV, sad: *M* = 0.78 μV; p < 0.001) and the parietal lobe (happy: *M* =-0.14 μV; neutral: *M* = 0.11 μV, sad: *M* = 0.60 μV; *p* < 0.001) (Figure 2).

#### P2 Component

The results of ANOVA showed that the main effect of emotion was not significant, *F*_(2, 80)_ = 0.21, *p* = 0.81, $\eta_{p}^{2}$ = 0.005. The main effect of group was significant, *F*_(1, 40)_ = 8.54, *p* = 0.01, $\eta_{p}^{2}$ = 0.176. The amplitude of the P2 wave in the depression group (*M* = 2.86 μV) was significantly higher than that in the normal group (*M* = 0.87 μV). The main effect of ROI was significant, *F*_(2, 80)_ = 16.53, *p* < 0.001, $\eta_{p}^{2}$ = 0.006. Further analysis showed that the P2 amplitude in the parietal lobe (*M* = 2.95 μV) was significantly larger than that in the central lobe (*M* = 1.70 μV; *p* < 0.001) and the frontocentral lobe (*M* = 0.94μV; *p* < 0.001). The three-way interaction was not significant, *F*_(4, 160)_ = 1.47, *p* = 0.23, $\eta_{p}^{2}$ = 0.035. The interaction between emotion and group was not significant, *F*_(2, 80)_ = 0.25, *p* = 0.71, $\eta_{p}^{2}$ = 0.006, the interaction between group and ROI was not significant, *F*_(2, 80)_ = 0.23, *p* = 0.65, $\eta_{p}^{2}$ = 0.006, and the interaction between emotion and ROI was not significant, *F*_(4, 160)_ = 0.64, *p* = 0.56, $\eta_{p}^{2}$ = 0.016.

#### N2 Component

The results of ANOVA showed that the main effect of emotion was significant, *F*_(2, 80)_ = 4.85, *p* = 0.01, $\eta_{p}^{2}$ = 0.108. The average amplitude of N2 components induced by sadness was higher than that induced by happiness. In addition, there were no significant differences in the average amplitudes of N2 components between sad and neutral emotional stimuli and between neutral and happy emotional stimuli (all *p* > 0.05). The main effect of group was not significant, *F*_(1, 40)_ = 1.75, *p* = 0.19, $\eta_{p}^{2}$ = 0.042. The main effect of ROI was significant, *F*_(2, 80)_ = 107.97, *p* < 0.001, $\eta_{p}^{2}$ = 0.730. Further analysis showed that the N2 amplitude in the frontocentral lobe (*M* = 3.65 μV) was significantly larger than that in the central lobe (*M* = 1.64 μV; *p* < 0.001) and the parietal lobe (*M* = −2.55 μV; *p* < 0.001). The three-way interaction was not significant, *F*_(4, 160)_ = 2.02, *p* = 0.13, $\eta_{p}^{2}$ = 0.048. The interaction between emotion and group was not significant, *F*_(2, 80)_ = 0.71, *p* = 0.49, $\eta_{p}^{2}$ = 0.017. The interaction between emotion and ROI was not significant, *F*_(4, 160)_ = 1.11, *p* = 0.34, $\eta_{p}^{2}$ = 0.027. The interaction between group and ROI was significant, *F*_(2, 80)_ = 5.86, *p* = 0.02, $\eta_{p}^{2}$ = 0.128. Further simple effect analysis showed that the average N2 amplitude evoked in the frontocentral lobe (depression group: *M* = 2.37 μV; normal group: *M* = 4.94 μV) was significantly higher than that in the central lobe (depression group: *M* = 2.37 μV; normal group: *M* = 4.94 μV) and the parietal lobe (depression group: *M* = −2.38 μV, normal group: *M* = −2.71 μV).

#### LPP Component

The mixed ANOVA with emotion, modality and group factors on the LPP components showed that the main effect of emotion was not significant, *F*_(2, 80)_ = 1.37, *p* = 0.26, $\eta_{p}^{2}$ = 0.033. The main effect of group was not significant, *F*_(1, 40)_ = 0.19, *p* = 0.66, $\eta_{p}^{2}$ = 0.005. The main effect of ROI was significant, *F*_(2, 80)_ = 103.74, *p* < 0.001, $\eta_{p}^{2}$ = 0.722. The average amplitude of LPP in the frontocentral lobe was significantly larger than that in the central lobe (*M* = −0.74 μV, *p* < 0.001) and the parietal lobe (*M* = −4.96 μV, *p* < 0.001). In addition, the average amplitude of LPP in the central lobe was significantly higher than that in the parietal lobe (*p* < 0.001). The three-way interaction was significant, *F*_(4, 160)_ = 34.05, *p* < 0.001, $\eta_{p}^{2}$ = 0.460. We then conducted a mixed-factors analysis of variance with 2 (group: depression group, normal group) ^*^ 3 (ROI: frontocentral lobe, central lobe, parietal lobe) factors with the three emotions. For the three emotions, the average amplitude of LPP evoked in the frontocentral lobe was significantly higher than that evoked in the central lobe and parietal lobe (all *p* < 0.001). In addition, the average amplitude of LPP in the central lobe was significantly higher than that in the parietal lobe (*p* < 0.001). In addition, after the analysis, it was found that in the sadness condition, the average amplitude of LPP in the frontocentral lobe in the depression group (*M* = 1.32 μV) was significantly higher than that in the normal group (*M* = 5.04 μV). There were no significant differences in the average amplitudes of LPP components between the two groups in the other ROIs. However, in the happy condition, there was no significant difference in the average amplitude of the LPP component between the two groups. The average amplitude of LPP evoked in the frontocentral lobe in the depression group (*M* = 0.81 μV) was significantly lower than that in the normal group (*M* = 3.12 μV). There were no significant differences in the average amplitudes of the LPP components between the two groups in the other ROIs.

**Figure 3:**
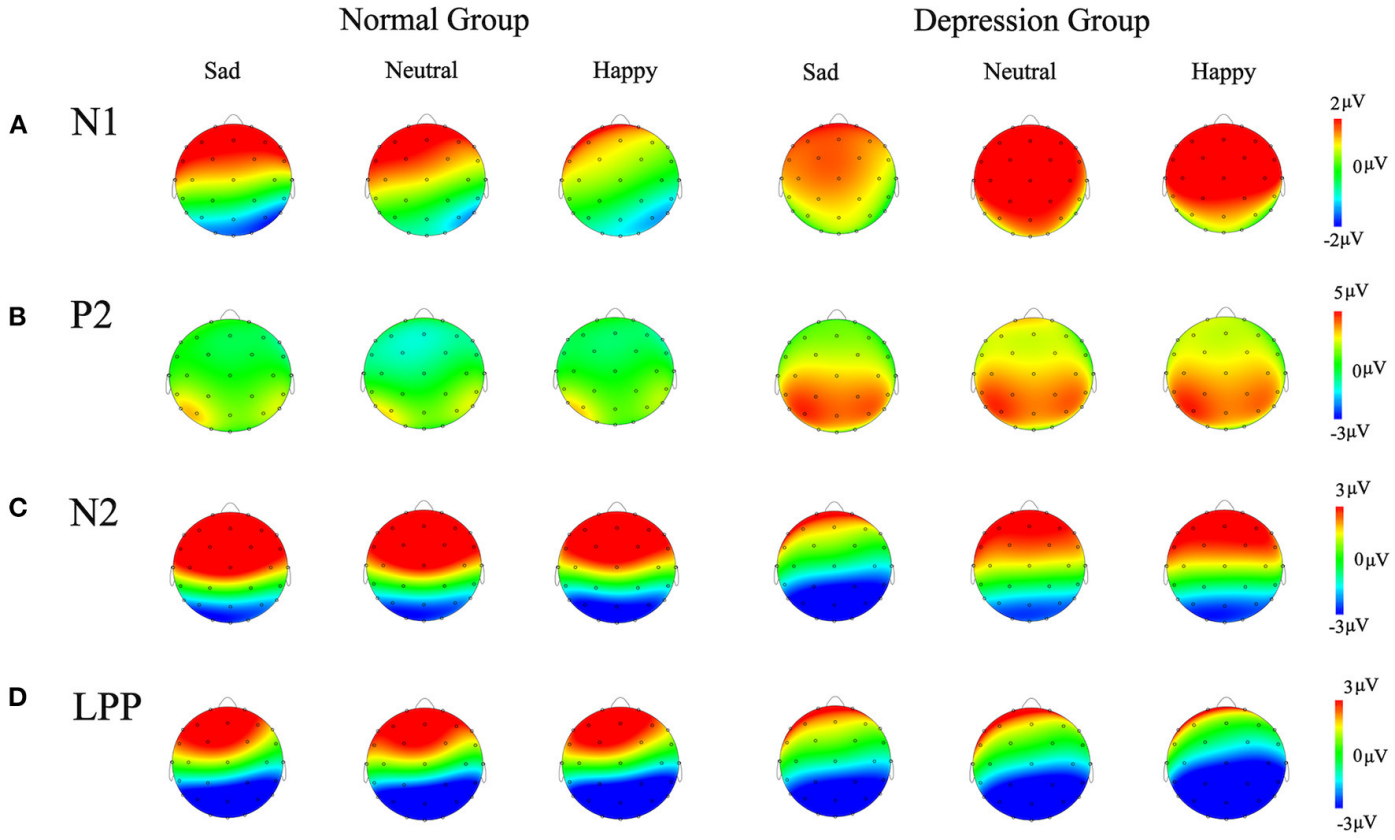
Topography maps of the difference waves **(A)** N1 component, **(B)** P2 component, **(C)** N2 component, **(D)** LPP component are shown in the different groups for different emotions.

To explain the differences in the sad emotional conditions, neutral emotional conditions and happy emotional conditions, we also carried out repeated analysis of variance with 3 (emotions: sad, neutral, happy) × 3 (ROI: frontocentral lobe, central lobe, parietal lobe) factors between the depression group and the normal group. The results showed that the main effects of emotion were significant in both the depression group and the normal group. The average amplitude of LPP induced by sadness (*M* = −1.09 μV) was significantly higher than that induced by happiness (*M* = −3.24 μV; *p* = 0.005). In addition, there was no significant difference in the average amplitudes of LPP induced by the other emotions, but the opposite results were found in the normal group. That is, in the normal group, the average amplitude of LPP induced by the sad emotional stimuli was significantly lower than that induced by the happy emotional stimuli (*M* = −0.65 μV; *p* = 0.001) and neutral emotional stimuli (*M* = −0.77 μV; *p* = 0.004). There was no significant difference in the average amplitudes of LPP induced by neutral emotional and happy emotional stimuli (Figure 3).

## Discussion

In this paper, the processing of visual, audio and audiovisual stimuli evoking happy, neutral and sad emotions from depressed participants was studied, and the event-related potential technique was used to study the effects of crossmodal integration of emotional information in these depressed participants. It was found that the participants had a higher hit rate and shorter response time to audiovisual target stimuli, and the response time and hit rate to emotional stimuli in the depression group were better than those in the normal group. In addition, the N2 amplitudes induced by sadness were significantly higher than those induced by happy emotions. Larger amplitudes of N1 and P2 were evoked in the participants in the depression group, and the average amplitude of LPP evoked in the frontocentral lobe in the depression group was significantly lower than that in the normal group.

First, the participants had higher hit rate and shorter response times to audiovisual target stimuli than to visual target stimuli or auditory target stimuli. This showed that individuals show an enhancement of crossmodal integration of emotional information when processing emotional information. This result is consistent with previous research results; that is, when the participants made emotional category judgments, voices consistent with the emotional valence of the face promoted processing of the face, which resulted in faster responses and higher hit rates (46). In addition, we found that the response times and hit rates to emotional stimuli in the depression group were better than those in the normal group, indicating that people with depression were more sensitive to emotional information. A number of studies have suggested that depression is linked with emotional reactivity (47–51). Emotional reactivity is the intensity, duration, and breadth of emotional experiences (52); thus, it is possible that depressed people were activated by the emotional stimuli to perform better in the task.

This ERP study found that cross-channel emotional facial expressions and sound stimuli induced smaller N1 waves than single-channel facial or sound stimuli. This is consistent with previous studies showing that audiovisual integration automatically occurs as early as 110 ms after stimulus presentation (30, 53); furthermore, in more complex presentation patterns (sound, facial expression and body posture), two-channel emotional information induced a smaller N1 amplitude than that through a single channel (54). This study also found that the participants in the depression group showed larger N1 amplitudes than those in the normal group. Since N1 reflects attention-related brain activity in the perceptual stage of early information processing (55), the results of this study may suggest that the depression group had weaker cognitive processing abilities in the early cognitive stages following stimulus presentation and needed more cognitive resources for processing than the normal group.

At the same time, this study found that the average amplitudes of P2 components with all emotional stimuli in the depression group were higher than those in the normal group. Some electrophysiological studies have shown that changes in P2 amplitudes are related to redundant, coherent and convergent processing of emotional information in dual channels (31, 33). Some studies have shown that P2 reflects the more refined processing of perceptual stimuli (56), and more excitation with emotional stimuli has been associated with higher P2 amplitudes (57). In addition, some researchers have studied patients with depression and found that sad faces induced larger P2 amplitudes in the experimental group, indicating that the increase in P2 amplitudes reflected the increased attention of individuals to sad faces (58). Therefore, in this study, the participants in the depression group showed a stronger P2 amplitude, probably because emotional stimuli were allocated more attentional resources in this group, and therefore, these stimuli were given priority processing.

Regarding the midterm component N2, we found that the amplitude of N2 induced by sadness was significantly higher than that induced by happiness. This result supports the “negativity-bias” framework of emotional processing (13, 59), in which negative bias is considered to be the result of rapid attentional resource allocation to negative stimuli. This rapid response to negative stimuli is adaptive to individuals and populations and can help people detect and avoid threatening situations more quickly (13). Therefore, individuals' attention is automatically directed toward threatening stimuli (60), but not positive stimuli, which gives processing priority to those threatening stimuli. In addition, some researchers believe that the stronger N2 amplitudes are, the stronger the active inhibition ability of the participants (37). Therefore, when the participants are faced with negative stimuli, they may automatically suppress them, thus inducing larger N2 amplitudes.

Finally, we found that the average amplitude of LPP evoked in the frontocentral lobe in the depression group was significantly lower than that in the normal group, which was consistent with previous studies; that is, in the field of emotional information processing, depressed patients and individuals with higher degrees of depression showed weaker LPP amplitudes to positive and negative stimuli (61–64). In addition, the amplitude of LPP produced by processing sad stimuli in the depression group was significantly larger than that produced by processing happy stimuli, while the opposite was true in the normal group, where the amplitudes of LPP induced by happy stimuli were larger. This may suggest that negative emotional stimuli induced higher arousal in patients with depression, thus inducing greater LPP amplitudes (15), which could allow individuals to devote more attentional resources to achieve fine coding of negative emotional stimuli (13). For example, some depression-related words or sad faces can well-reflect patients' negative attention bias (65). In addition, studies have shown that LPP can reflect the intensity of motivation, and desired stimuli associated with higher motivation intensities can induce greater LPP amplitudes (66). Studies have shown that anxiety patients watching angry faces and scary faces show greater LPP amplitudes (63). Therefore, in this study, the depressed participants showed greater LPP amplitudes in response to sad stimuli. These data show that negative stimuli can induce stronger emotional and motivational responses in depressed individuals; that is, depressed participants have a stronger motivation to approach negative stimuli. In contrast, the participants in the normal group had larger LPP amplitudes to happy stimuli, indicating that normal individuals had a stronger motivation to approach positive stimuli.

In conclusion, our results showed that individuals show the effects of crossmodal integration of emotional information while processing emotional stimuli, and there was a difference in the effects of emotional stimuli between the depression group and the normal group. The difference in this integration effect is of great significance for us to understand the processing bias to negative stimuli in people with depression. We can speculate that this abnormal processing of emotional information may lead to the abnormal development of social cognition in patients with depression. Therefore, problems related to social emotional abilities that have been observed in patients with depression are not entirely caused by the social environment. Early information processing components may also play an important role. Therefore, based on these results, we suggest that future studies apply more naturalistic experimental designs to investigate emotional and social deficits in depression. These findings also have certain significance regarding the exploration for potential treatment options for patients with depression; for example, in future intervention programs, we can consider the training of crossmodal integration with personalized emotional stimuli.

## Data Availability Statement

The raw data supporting the conclusions of this article will be made available by the authors, without undue reservation.

## Ethics Statement

The studies involving human participants were reviewed and approved by the Ethics Committee of Hubei University. The participants provided their written informed consent to participate in this study.

## Author Contributions

TL and XZ wrote the manuscript. YC, XZ, ZG, and NW performed the experiments. JY, SL, and WY analyzed the data. TL, WY, and SL conceived and designed the experiments. JY, WY, ZG, and NW revised the manuscript and approved the final version. All authors contributed to the article and approved the submitted version.

## Conflict of Interest

The authors declare that the research was conducted in the absence of any commercial or financial relationships that could be construed as a potential conflict of interest.
